# Optical and Mechanical Characteristics of One-Shade Composite Resins

**DOI:** 10.3390/jfb16110419

**Published:** 2025-11-08

**Authors:** Jee Eun Shim, Hyun-Jung Kim, Soram Oh, Ji-Hyun Jang

**Affiliations:** 1 Department of Conservative Dentistry, Graduate School, Kyung Hee University, Seoul 02447, Republic of Korea; pepe0929@gmail.com; 2 Department of Conservative Dentistry, School of Dentistry, Kyung Hee University, Seoul 02447, Republic of Korea; kimhyunjung@khu.ac.kr (H.-J.K.); soram@khu.ac.kr (S.O.)

**Keywords:** one-shade composite resin, bulk-fill resin, shade, color adjustment, microhardness, mechanical property

## Abstract

This study evaluated the optical and mechanical properties of two single-shade composite resins compared with a conventional multi-shade composite. Omnichroma (OM), Metafil Bulk Fill ONE (BO), and Filtek Z350XT (Z350) were tested. Color adjustment was assessed using A3, B1, and C4 background cavities, and ΔE00 values were calculated. The translucency parameter (TP) was measured, and the flexural strength, flexural modulus, and depth of cure (B/T ratio) were determined. OM and BO showed better color adjustment performance on brighter (B1) backgrounds and decreased matching on darker (C4) ones. OM maintained stable color adjustment across cavity depths, while BO showed improved adjustment in shallower cavities. Both exhibited higher TP values than Z350. The control group (Z350) had the highest flexural strength and modulus, though BO’s flexural strength was comparable. OM and BO showed sufficient mechanical strength and a greater depth of cure compared to Z350. Our study indicated that the one-shade composite resins OM and BO exhibited better color adjustment performance compared to conventional composite resins due to the influence of the surrounding shades, with a better adjustment ability on brighter backgrounds. Additionally, OM and BO demonstrated sufficient strength and a higher depth of cure compared to the control group.

## 1. Introduction

The esthetic requirements placed on composite resins are intimately linked to the optical interaction between materials and light. Because the polychromatic nature of teeth—such as the age, size, and tooth type—influences the tooth color, it is important to match these colors before placing a restoration or repairing an existing one [1]. The dental market has offered a variety of direct resin systems, presenting variable shade options along with diverse polychromatic natured teeth and previous restorative materials’ shades [2,3].

Conventionally, the shades of teeth and composite resins have been determined visually using the VITA Classical Shade Guide system (Vita, Zahnfabrik, Sackingen, Germany), with most commercially available resin composites offering 16 shades, from A1 to D4 [3,4]. However, discrepancies in color between shade guides, composite resins, and natural teeth often result in suboptimal shade matching. In addition, the perceived color of the final restoration is affected by the inherent darkness of the oral cavity, which acts as the background. To address these limitations, various shades and opacities of resin composites have been utilized in multilayering techniques to reproduce natural tooth characteristics. Nevertheless, the success of such restorations depends on precise shade selection and a high level of technical expertise, relying heavily on the clinician’s experience and skill [5,6].

The observed alteration in coloration at the periphery of a restored resin composite depends on the mutual reflection of color between the adjacent tooth structure and the restoration. This color blending effect at the border could be related to the properties of light transmission and diffusion within the resin composite, the enamel prism orientation at the border, the composition of the enamel margin, and the age of the restored tooth [2,6,7]. Ismail EH. and others reported that the color of the surrounding environment might affect the color of the resin composite by as much as 39% when compared to the resin composite without any surroundings [8]. The phenomenon of color transition at the margin of a restored resin composite is commonly referred to as the “chameleon effect” (blending effect), and it makes the restoration more esthetic, simplifying the number of shades [2,6].

This property has led to the development of novel dental composites, simplifying shade selection and enhancing reproducibility. Recently, the concept of “single-shade” or “one-shade” composites has been introduced. Manufacturers claim that these composites can match various multi-VITA scale shades, and recent investigations have demonstrated the color-blending effect of the one-shade resin [3,8]. The advantage of one-shade resin composites is their excellent color adjustment potential, which refers to the interplay of perceptual and physical elements. Omnichroma (OM; Tokuyama Dental, Tokyo, Japan) is the first-introduced one-shade resin composite. According to the manufacturer, OM is pigment-free and contains 260 nm uniform spherical fillers that reflect the yellow-to-red wavelength range, contributing to the structural color formation and color adjustment ability. Demonstrating exceptional color-matching ability, it covers the entire range of VITA classical shades with just one composite shade [6,7,8,9].

Bulk-fill resin composites were also developed to simplify the restorative process. They aimed to accelerate the restoration process by allowing thick increments of up to 4–5 mm to be cured at once, by increasing the transparency of the resin, enabling polymerization to occur in one step [10]. Bypassing the time-consuming layering process, it could be characterized by significantly reduced shrinkage stresses and polymerization contraction [10,11,12,13,14]. Metafill Bulkfill ONE (BO; Sun Medical, Moriyama, Japan) is the most recently developed one-shade and bulk-fill resin.

Recent studies have investigated the color adjustment ability and mechanical properties of one-shade resin composites [5,6,7,8,9]. Although manufacturers claim that a single shade can match the entire range of VITA Classical shades, evidence regarding their performance across diverse background shades and material thicknesses reported that conventional multi-shade composites usually achieve a better color matching performance in in vitro studies. On the other hand, comprehensive assessments of both the mechanical performance and aesthetic outcomes of BO, which is the one-shade composite with the bulk-fill concept, have been rarely examined.

In this study, we evaluate the color adjustment ability of two different one-shade resins to various background shades with different thicknesses and evaluate their mechanical properties. A color adjustment test and translucency measurement test were performed to assess the optical behavior of one-shade resins, and, to evaluate their mechanical properties, flexural strength, elastic modulus, and depth of cure tests were conducted.

The null hypotheses evaluated in this study were as follows: (1) One-shade resin composites will show no difference in color adjustment ability according to the different background shades and cavity depths. (2) There would be no differences in color adjustment ability between the one-shade resin and conventional multi-shade resins. (3) There would be no differences in mechanical properties between the one-shade resin and conventional multi-shade resins.

## 2. Methods and Materials

### 2.1. Color Adjustment Potential Analysis

**Figure 1:**
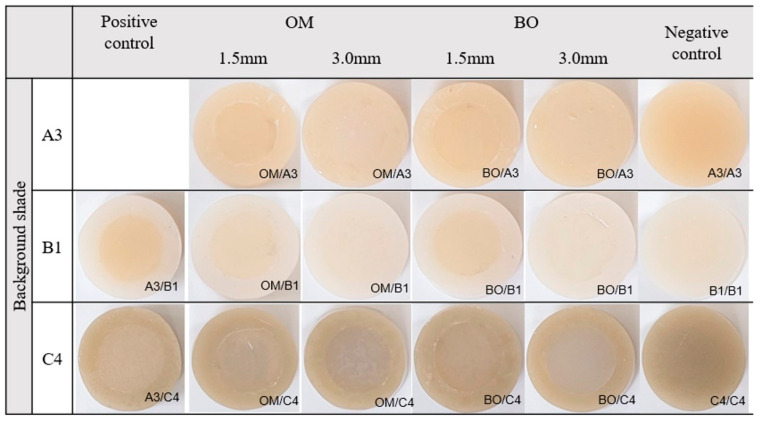
Representative images of the specimens for color adjustment analysis. In the left column (positive control), the inner cavities were filled with Z350 shade A3 on backgrounds of three different shades (A3, B1, and C4). In the right column (negative control), the inner cavities were filled with Z350 matching the shade of each corresponding background (A3, B1, and C4). The experimental one-shade resin composites, Omnichroma (OM) and Metafil Bulk Fill ONE (BO), were filled into the inner cavities with various depths and background shades (A3, B1, and C4).

A total of fifty-one disk-shaped (10 mm in diameter and 5 mm in height) composite resin specimens were prepared using a stainless-steel mold. To create an inner concave cavity, a removable composite resin insert (6 mm in diameter, 1.5 or 3 mm in thickness) placed with Teflon tape was positioned at the center of the mold before curing. The outer background disks were fabricated using three shades (A3, B1, and C4) of a conventional resin composite, Filtek Z350XT (Z350; 3M ESPE, St. Paul, MN, USA). After light curing, the insert was carefully removed to obtain an outer disk specimen with a concave inner cavity. Then, the inner cavities were restored with two one-shade resins, OM and BO low-flow (BO; Sun Medical, Moriyama, Japan), while Z350 was used as the positive and negative controls. Detailed information on the materials used in this study is provided in Table 1. A transparent polyester strip and glass slide were placed over the top of each sample and cured under an LED device (Elipar Deep Cure-S LED curing light; 3M ESPE, St. Paul, MN, USA) at the intensity of 1470 mW/cm^2^ for 20 s. The resin disks were stored for 24 h in distilled water and darkness at room temperature. Then, they were finished using 600-grit SiC paper under running water to prevent irregular reflections from the surface. The representative samples of groups are shown in Figure 1.

The color parameters (L*, a*, and b*) were determined based on the Commission International de L′eclairage (CIE) with a VITA Easyshade V spectrophotometer (VITA Zahnfabrik, Bad Sackingen, Germany) over a white background for 5 times for each sample. The VITA Easyshade V was calibrated before each sample measurement according to the manufacturer’s protocol by placing the probe tip on the calibration port aperture. A standard white ceramic calibration tile was used to verify the accuracy and stability of the device. Measurements were taken by positioning the probe tip as flat as possible to the surface of the sample. The color of each specimen was measured on the surface of the one-shade composite resin in the inner cavity, with the surrounding multi-shade resin background.

The color adjustment potential was defined as the color difference (ΔE) between the one-shade composite resin in the inner cavity and the surrounding multi-shade resin background, and it was calculated using the following equation, Equation (1), according to the CIEDE2000 (ΔE_00_) formula [15]. This color difference formula, grounded in the CIELAB color space, is designed to correct the discrepancies between measurement outcomes and visual assessment, and is more sensitive than the CIELAB color difference (ΔE_ab_).

(1) $${∆E}_{00}=\sqrt{{\left(\frac{∆L^{\prime }}{{K_{L}S}_{L}}\right)}^{2}+{\left(\frac{∆C^{\prime }}{{K_{C}S}_{C}}\right)}^{2}+{\left(\frac{∆H^{\prime }}{{K_{H}S}_{H}}\right)}^{2}+R_{T}\left(\frac{∆C^{\prime }}{{K_{C}S}_{C}}\right)\left(\frac{∆H^{\prime }}{{K_{H}S}_{H}}\right)}$$

The differences in lightness (ΔL), chroma (ΔC), and hue (ΔH) are denoted. The rotation function (R_T_) takes into account interactions between hue and chroma differences in the specified region. S_L_, S_C_, and S_H_ are the weighting functions for modifying the overall color difference in the L′, a′, and b′ coordinates. The parametric factors (K_L_, K_C_, and K_H_) serve as correction terms for experimental conditions.

### 2.2. Translucency Measurement

The three disks (diameter 10 mm, height 2 mm) of each experimental (OM, BO) and control (Z350 A3) resin composite were prepared. The top of each sample was covered with a transparent polyester strip and glass slide and cured under an LED device (Elipar Deep Cure-S LED curing light; 3M ESPE, MN, USA) for 20 s at the intensity of 1470 mW/cm^2^. The resin disks were polished using 600-grit SiC paper under running water to avoid irregular reflections from the surface. After curing, the samples were stored in a dark environment at room temperature in distilled water for 24 h.

The color parameter is determined using the CIELAB color scale over a black and white background for the same sample, with a VITA Easyshade V spectrophotometer 5 times.

The translucency of the samples was assessed by determining the color difference between the sample against a black and white background, utilizing the following CIEDE2000 color difference formula equation, Equation (2) (TP_00_) [16]:

(2) $${TP}_{00}=\sqrt{{\left(\frac{{L^{\prime }}_{B}-{L^{\prime }}_{W}}{{K_{L}S}_{L}}\right)}^{2}+{\left(\frac{{C^{\prime }}_{B}-{C^{\prime }}_{W}}{{K_{C}S}_{C}}\right)}^{2}+{\left(\frac{{H^{\prime }}_{B}-{H^{\prime }}_{W}}{{K_{H}S}_{H}}\right)}^{2}+R_{T}\left(\frac{{C^{\prime }}_{B}-{C^{\prime }}_{W}}{{K_{C}S}_{C}}\right)\left(\frac{{H^{\prime }}_{B}-{H^{\prime }}_{W}}{{K_{H}S}_{H}}\right)}$$

The subscripts “B” and “W” denote the lightness (L′), chroma (C’), and hue (H′) of the specimens over black and white backgrounds, respectively. R_T_ represents the rotation function, accounting for interactions between hue and chroma differences in the specified region. The parametric factors (K_L_, K_C_, and K_H_) are used to adjust for experimental conditions. S_L_, S_C_, and S_H_ are the weighting functions for modifying the overall color difference against the black and white backgrounds.

### 2.3. Flexural Strength and Flexural Modulus

Based on the ISO 4049:2019 specification, samples (*n* = 15/group) with dimensions of 25 × 2 × 2 mm (length × width × height) were fabricated for both the experimental groups (OM and BO) and the control group (Z350 A3) [17]. For the build-up of each sample, a mold was covered with a transparent polyester strip and glass slide on top and cured for 20 s with an LED device (Elipar Deep Cure-S LED curing light, 3M ESPE) at the intensity of 1470 mW/cm^2^. After removing the strip, additional polymerization was carried out for 20 s on the bottom of the samples. After curing, samples were finished using 600-grit SiC papers under running water and stored in distilled water at room temperature for 24 h.

To determine the elastic modulus (E) and flexural strength (σ) of the materials, a universal testing machine (AGS-X, Shimadzu, Japan) was used. In a three-point bending setup, the samples were tested until failure, with a 20 mm spacing between the supports and a crosshead speed of 0.75 mm/min. The maximum loads were recorded, and the flexural strength in MPa and elastic modulus in GPa were calculated using the following formulae, Equation (3) [18]:

(3) $$\;E=\frac{FL^{3}}{4BH^{3}d}\;\;\;\;\;\;\;\;\;\sigma =\frac{3FL}{2BH^{2}}$$

Subscripts “F”, “L”, “B”, “H”, and “d” refer to the maximum load (N), support span (mm), sample width (mm), height (mm), and deflection (mm), respectively.

### 2.4. Depth of Cure

The five disks (diameter 4 mm, height 4 mm) of each experimental (OM and BO) and control (Z350 A3) resin composite were prepared in molds placed on glass slides. The mold was filled according to the manufacturer’s instructions, with multiple increments (2 mm each, applied twice) for Z350 A3 and OM samples, while a single increment was used for the BO samples. A transparent polyester strip and glass slide were covered on top of the mold and light-activated for 20 s with an LED device (Elipar Deep Cure-S LED curing light, 3M ESPE) at the intensity of 1470 mW/cm^2^. After curing, samples were stored in dry, room temperature for 24 h.

The depth of cure was determined using the Vickers microhardness ratio method. The Vickers microhardness of the top and bottom surfaces of each specimen was measured using a microhardness tester (HMV-G Series, Shimadzu, Japan) under a 4.903 N load for 5 s. Three indentations were made on each surface, and the bottom-to-top (B/T) hardness ratio (%) was calculated. A B/T ratio of 0.8 or higher was considered indicative of an adequate polymerization depth.

### 2.5. Statistical Analysis

The data was analyzed by one-way analysis of variance (ANOVA) using SPSS version 25 (SPSS, IBM Corp., Armonk, NY, USA) followed by Tukey HSD post hoc test and Scheffe correction. All procedures were conducted at a significance level of *p* < 0.05.

## 3. Results

### 3.1. Color Adjustment Analysis

**Figure 2:**
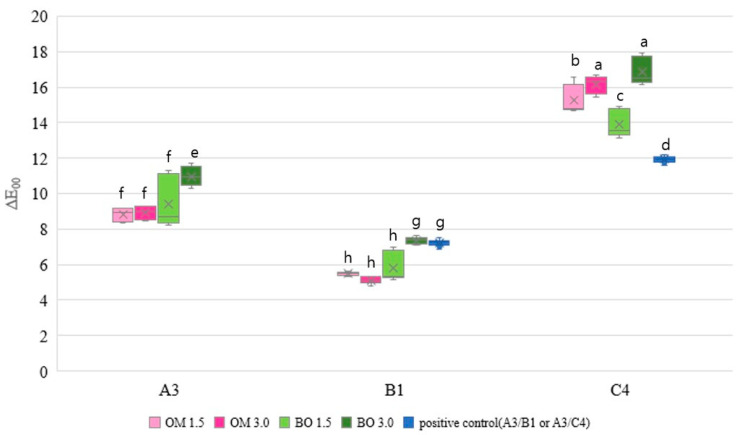
Color differences (ΔE_00_) of resin composites filled in various background shades and depths of the cavity. The 50:50% perceptibility threshold and acceptability threshold are 0.8 and 1.8, respectively. Different letters indicate significant differences (*p* < 0.05).

The representative images of specimens for color adjustment are presented in Figure 1. The average values and standard deviations of the color parameter measurements and color differences for various cavity thicknesses and background shades are presented in Table 2 and Figure 2.

In the background shade A3, OM demonstrated a consistent color adjustment ability regardless of the thickness of the composite, whereas BO exhibited a pronounced ΔE_00_ increase at a depth of 3 mm. No significant difference was observed between BO 1.5 and OM across all cavity depths (*p* < 0.05).

In background shade B1, similar trends were observed as in the A3 shade background. OM showed no variation with thickness, while BO exhibited a high ΔE_00_ at 3 mm. Except for BO 3.0, all groups exhibited a lower ΔE_00_, suggesting superior color matching compared to the positive control, which restored the cavity with an A3 shade (*p* < 0.05).

In the background shade C4, both OM and BO showed a higher ΔE_00_ in comparison to the positive control. Interestingly, as the cavity depth decreased, a corresponding reduction in thickness led to a decrease in ΔE_00_, suggesting improved color adjustment (*p* < 0.05).

**Figure 3:**
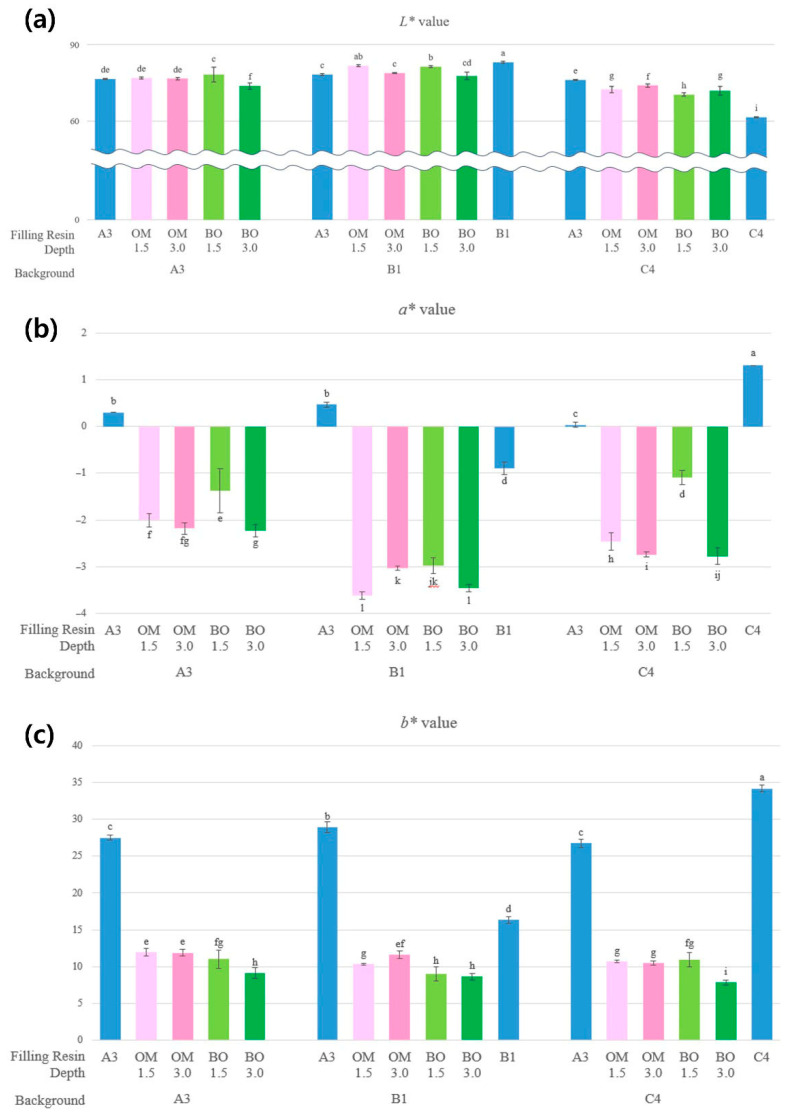
(**a**) Lightness (L*), (**b**) green–red value (a*), and (**c**) blue–yellow value (b*) of resin composites filled in various background shades and depths of the cavity. Different letters indicate significant differences (*p* < 0.05).

Each color parameter (L*, a*, b*) displayed its unique trend, as shown in Table 2 and Figure 3.

### 3.2. Translucency Measurement

The TP values of samples are presented in Table 3. The TP values showed significant differences between groups (*p* < 0.05). Both OM and BO exhibited a higher ΔTP_00_ compared to the control group, with OM showing a higher value (*p* < 0.05).

### 3.3. Flexural Strength and Flexural Modulus

The results of the flexural strength and elastic modulus are presented in Table 4. The control group showed 139.78 MPa of flexural strength, which is the highest of the samples, but no significant difference was observed between BO and the control group, as well as between OM and BO (*p* > 0.05).

The flexural modulus was significantly higher in the control group (11.48 GPa) compared to the experimental groups, with the following order showing significant differences between each value: control > OM > BO (*p* < 0.05).

### 3.4. Depth of Cure

**Figure 4:**
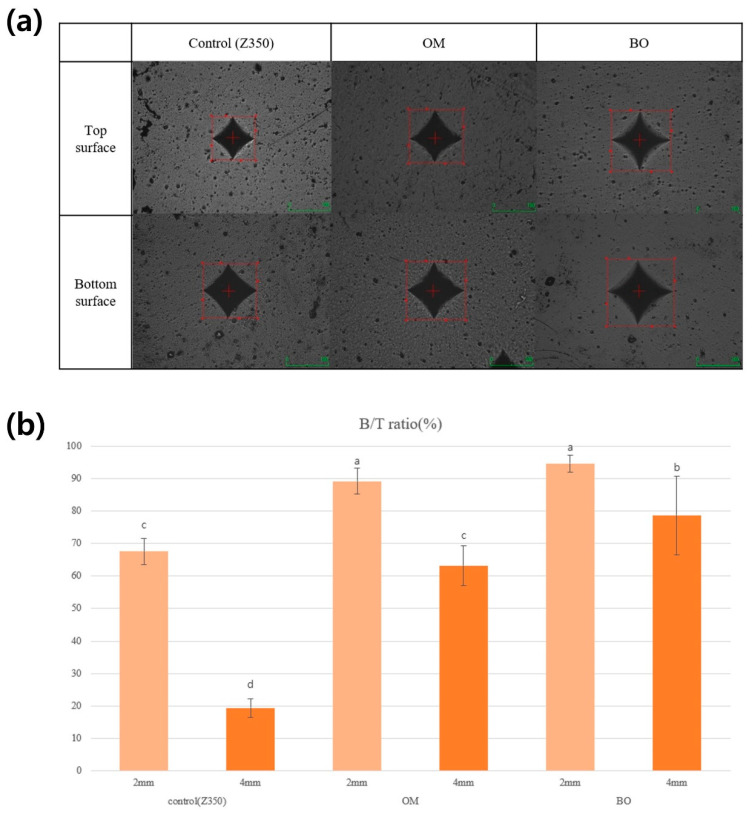
(**a**) Representative images of Vickers microhardness indentation of top and bottom surfaces and (**b**) bottom-to-top (B/T) ratio of the resin composites. Different letters indicate significant differences (*p* < 0.05).

Representative images obtained by testing the top and bottom surfaces of each sample with a Vickers diamond indenter are shown in Figure 4a and the results of the Vickers microhardness and bottom-to-top ratio (B/T ratio) are presented in Table 5.

Significant differences in the B/T ratio are observed between groups (*p* < 0.05) (Figure 4b). The experimental group demonstrated a superior depth of cure compared to the control group. OM exhibited the highest B/T ratio followed by BO (*p* < 0.05).

## 4. Discussion

In this study, while OM did not show significant ΔE_00_ differences concerning thickness on light background shades, BO exhibited a higher ΔE_00_ with increasing thickness across all background shades, indicating the differences compared to the control group. Additionally, the color adjustment ability of both OM and BO was found to be lower than that of the control group in a dark shade. According to these results, the first and second hypotheses were rejected.

The color adjustment ability can be quantified by ΔE_00_ and evaluated by the perceptibility threshold and acceptability threshold. The 50:50% perceptibility threshold and acceptability threshold values in dentistry are 0.8 and 1.8, respectively [19,20]. These values are significantly lower compared to those of ΔE_00_ in our study, indicating that they are clinically unacceptable. The mean ΔE_00_ values ranged between 5.10–7.37 on bright backgrounds (B1) and increased to 13.88–16.16 on dark backgrounds (C4). This discrepancy can be attributed primarily to the in vitro study design. The surrounding substrates used in this experiment were standardized color disks, which do not fully replicate the optical complexity of natural teeth. In the clinical situation, chromatic blending and subsurface light scattering through dentin and enamel often contribute to reduced color differences, enhancing the visual blending effect. Thus, while the ΔE_00_ values obtained in this study are numerically higher than the commonly accepted thresholds, they likely overestimate the perceived mismatch that would occur in clinical circumstances.

Despite these limitations, the relative trend among materials remains clinically relevant. Both OM and BO demonstrated superior color adjustment compared with the multi-shade control (Z350), particularly on brighter backgrounds, consistent with their high translucency and light-scattering formulations. Natural teeth are characterized by multiple colors, layers, and translucency which influence light reflection and scattering. However, since the specimens to which the one-shade resin composites were applied were not natural teeth, it can be inferred that the color adjustment ability was limited. In contrast to our study, the previous literature demonstrated acceptable levels of ΔE, which can be attributed to the smaller diameter and shallower cavities of the samples evaluated, accounting for the difference in the results [6]. The blending effect occurs when colors are perceived to be closer together when viewed collectively rather than individually [20]. Therefore, the remaining structure and intrinsic properties of a tooth may be an influential factor in the color adjustment ability.

According to a study that arranged the VITA classic shade guide by value, the control group using Z350 shades selected the brightest value (B1), the darkest value (C4), and the common natural tooth color (A3) to represent typical dental shades [21]. The trend in the results of our study showed the best color adjustment for the experimental group in background B1 (Figure 2). With the transition of the background shade from B1 to C4, ΔE showed a corresponding increase. In the previous literature describing results similar to those of this study, the composite resin exhibited the capacity to adjust its shade in response to the brightening of the surrounding tooth structure [2,6,22]. Therefore, it was suggested that multi-shade composite systems would be advantageous in darker background shades where higher esthetics are required.

In previous studies, the ΔE_00_ of OM increased in the deeper cavity indicating a decrease in color adjustment [7,23]. However, in our research, as the thickness increased, the color adjustment ability of OM was maintained, whereas BO exhibited a significant decrease across all background shades. This observed phenomenon may be attributed to the content, shape, and type of fillers. Arikawa H. et al. reported that variations in the size and morphology of fillers incorporated into the materials may result in significant variances across the materials in the distribution spectrum of transmitted light [24]. On the basis, in BO, the combination of a low filler content and small irregular filler shapes enhances the light transmittance, thereby influencing both the structural color and color adjustment ability. This reduced filler content in BO, compared to OM, notably affects the spectral reflectance, leading to a noticeable decrease in color adjustment ability, particularly in deep cavity conditions [25].

The color parameter L* values exhibited variations according to the background shade (Figure 3a). Higher L* values of the one-shade resin were observed under bright backgrounds, while lower values were observed under dark backgrounds. In contrast, as shown in Figure 3b,c, the color parameter a* and b* values of one-shade resin composites exhibited a clustered distribution in a specific range, with a* ranging from −3.62 to −1.09 and b* ranging from 7.82 to 11.93. The one-shade resin composite’s a* and b* values were considerably lower compared to the control group across all background shades and thicknesses. This indicates that the one-shade resin itself exhibits a stronger tendency towards bluish and greenish color [26]. In particular, BO exhibited a more pronounced trend as the thickness increased, which appears to be associated with larger variations in ΔE.

Translucency is a significant optical property of resin composites. By scattering light upon transmission, it induces a chameleon effect, reflecting the color of the adjacent teeth and imparting its own color to nearby tooth structures [27,28]. Therefore, we aimed to compare the translucency of the control and experimental groups to assess their color adjustment performance. According to Table 2, the highest results were observed in OM, and this might be due to the consistent size and shape of the filler system comprising OM, as previously mentioned in the color adjustment ability [20]. The lower scattering and absorbance of OM reduces the energy attenuation or loss within the material, thus justifying its superior translucency, which contrasts with the irregular filler shape found in other materials [5]. Given that the blending effect increases with the translucency parameter, based on the results of the earlier evaluation of color adjustment, it is believed that OM and BO exhibit superior color adjustment compared to the control group in background shades B1 and A3 [20,23].

Furthermore, this study evaluated the mechanical properties of the one-shade resin composites compared to conventional resins, since the physical properties of the resin composites also impact their characterization, like their clinical performance [29]. From these results, the third null hypothesis for the mechanical properties was partially rejected. The flexural strength and flexural modulus of the one-shade resin composites exhibited slightly lower values compared to those of the conventional resin composite. However, the flexural strength did not exhibit significant differences, while the depth of cure demonstrated excellent results.

The diversity in flexural properties among different resin composites is valuable across various clinical situations. Flexural strength denotes the maximum stress that resin composites can endure before reaching failure, and this property, along with other mechanical characteristics, varies depending on the filler content, shape, and type [13,30,31], as well as the composition and polymerization network formed within the resin-based composites during light activation [13,32]. Previous studies have demonstrated that increasing the volume percentage of fillers can enhance the flexural strength [33]. In this study, the flexural strength increased in the order of OM, BO, and Z350, and the flexural modulus increased in the order of BO, OM, and Z350, while the filler content was the highest in OM, followed by Z350 and BO (Table 4). Although OM contains a filler loading of approximately 79 wt%, 68 vol%, which, according to the manufacturer’s information, is comparable to that of Z350 (78.5 wt%, 63.3 vol%), several material-related factors contribute to the lower flexural strength of OM. First, it could be inferred that the shape and type of fillers contained in OM influenced the results inconsistent with the previous literature. Z350 employs nanosized zirconia/silica cluster fillers, which provide a more effective stress-transfer network and crack deflection under bending stress. In contrast, OM uses uniform spherical fillers (~260 nm SiO_2_–ZrO_2_ particles) designed primarily to optimize a broad light-scattering profile for color adjustment rather than mechanical reinforcement. Interestingly, BO, despite having a lower filler loading (74 wt%, 54.3 vol%), showed higher FS than OM, likely due to its bulk-fill formulation that allows deeper polymerization and may result in higher cohesive strength. And it is centered around irregular fillers rather than spherical fillers of OM, exhibiting a higher strength. However, its lower filler content explains the reduced flexural modulus, which corresponds with the findings from the previous literature comparing bulk-fill and conventional resin composites [11]. Second, the resin matrix composition and filler–matrix interfacial coupling might be different between the three experimental composites. OM contains a relatively higher proportion of low-viscosity monomers to improve translucency and handling, which can lead to a lower degree of polymer crosslinking and weaker interfacial adhesion.

The depth of cure was measured with an indirect method, using microhardness and the B/T ratio in this study. OM showed the highest value, followed by BO and Z350 (Figure 4 and Table 5). This can be attributed to the penetration of light into resin composites, as a higher translucency allows more curing light to enter and polymerize the composite evenly [11,13,34]. Moreover, Z350 and OM comply with the manufacturer’s recommendations for a light-curing depth of 2 mm, whereas BO demonstrated suitability for filling cavities up to 4 mm thick in a single layer. This method simplifies restorative procedures, saving clinical time for deep and wide cavities, and decreasing the sensitivity to operator-dependent factors [34].

In microhardness testing, the indentation size effect may influence the microhardness measurement, and it can lead to higher hardness values. Various factors such as the load magnitude, dwell time, indenter geometry, crosshead speed, and material’s elasticity recovery might influence the indentation size effect. In this study, the dwell time of five seconds was applied, which was consistent with our previous study [13,35]. Short dwell times may lead to the partial elastic recovery of the indentation, whereas excessively long dwell times may cause creep or time-dependent deformation that does not reflect clinical conditions [36,37]. Therefore, the potential influence of the short dwell time should be acknowledged when interpreting the hardness results.

In this in vitro study, the one-shade resin composite demonstrated sufficient physical properties for clinical application and exhibited an excellent color adjustment ability under specific conditions. In actual clinical scenarios, it is essential to recognize that the color adjustment ability is influenced not only by the inherent characteristics of the composite resin but also by the amount of remaining tooth structure, including the geometric configuration and dimensions of the cavity. These factors are crucial considerations in evaluating the performance and esthetic outcomes of resin composites. In this experiment, we evaluated only the impact of the cavity depth. However, it is hypothesized that variations in the cavity diameter could also influence the ΔE value. Therefore, further research is required to comprehensively understand the effects of cavity dimensions on the color adjustment ability and overall performance of resin composites.

## 5. Conclusions

One-shade composite resins are influenced by the surrounding shades, hence exhibiting a higher color adjustment ability compared to conventional composite resins, especially showing superior performance with brighter background shades. However, they exhibited material-dependent results regarding the cavity thickness. Furthermore, one-shade composite resins demonstrated sufficient strength compared to the control group, and they also exhibited a high degree of polymerization depth.

## Figures and Tables

**Table 1:** The resin composite materials used in this study.

Resin Composite (Group)	Manufacturer	Filler and Monomer Composition	Filler Content	Lot No.
			(wt/vol%)	
Filtek Z350XT (Z350)	3M ESPE, St. Paul, MN, USA	non-agglomerated/non-aggregated 20 nm silica filler, non-agglomerated/non-aggregated 4 to 11 nm zirconia filler, aggregated zirconia/silica cluster filler (comprising 20 nm silica and 4 to 11 nm zirconia particles) Bis-GMA *, UDMA **, TEGDMA ***, Bis-EMA †, PEGDMA ††	78.5/63.3	9783185
Omnichroma (OM)	Tokuyama Dental, Tokyo, Japan	260 nm SiO_2_-ZrO_2_ supra-nano spherical filler UDMA, TEGDMA	79/68	037E01
Metafill Bulkfill ONE (BO)	Sun Medical, Moriyama, Japan	Silane-treated barium silica glass UDMA, Bis-EMA	74/54.3	23071

* BisGMA bisphenol A diglycidildimethacrylate, ** UDMA urethane dimethacrylate, *** TEGDMA triethylene glycol dimethacrylate, † Bis-EMA Ethoxylatedbisphenol A dimethacrylate. †† PEGDMA polyethylene glycol dimethacrylate.

**Table 2:** Mean and standard deviation of color differences (ΔE_00_) and color parameters based on the application depth of OM and BO.

Cavity	L*	a*	b*	C*	H*	ΔE_00_
**Background shade A3**						
A3	76.58 (0.17) ^de^	0.30 (0) ^b^	27.52 (0.33) ^c^	27.52 (0.33) ^b^	89.36 (0.05) ^g^	-
OM 1.5	76.95 (0.44) ^de^	−2.01 (0.14) ^f^	11.93 (0.48) ^e^	12.09 (0.46) ^e^	99.54 (1.05) ^d^	8.82 (0.35) ^f^
OM 3.0	76.70 (0.49) ^de^	−2.18 (0.13) ^fg^	11.87 (0.44) ^e^	12.08 (0.44) ^e^	100.53(0.98) ^d^	8.94 (0.34) ^f^
BO 1.5	78.36 (2.90) ^c^	−1.37 (0.48) ^e^	11.02 (1.24) ^fg^	11.13 (1.17) ^f^	97.52 (3.56) ^e^	9.41 (1.32) ^f^
BO 3.0	73.84 (1.21) ^f^	−2.23 (0.13) ^g^	9.11 (0.69) ^h^	9.41 (0.64) ^g^	103.87 (1.92) ^c^	10.98 (0.49) ^e^
**Background shade B1**						
A3	78.34 (0.43) ^c^	0.47 (0.05) ^b^	28.91 (0.73) ^b^	28.91 (0.73) ^a^	89.08 (0.06) ^g^	7.22 (0.19) ^g^
OM 1.5	81.85 (0.38) ^ab^	−3.62 (0.09) ^l^	10.35 (0.11) ^g^	10.98 (0.10) ^f^	109.25(0.51) ^b^	5.48 (0.09) ^h^
OM 3.0	78.89 (0.20) ^c^	−3.03 (0.05) ^k^	11.63 (0.53) ^ef^	12.02 (0.52) ^e^	104.71 (0.67) ^c^	5.10 (0.19) ^h^
BO 1.5	81.48 (0.39) ^b^	−2.97 (0.17) ^jk^	9.00 (0.93) ^h^	9.49 (0.82) ^g^	108.51 (2.89) ^b^	5.81 (0.09) ^h^
BO 3.0	77.82 (1.40) ^cd^	−3.46 (0.08) ^l^	8.63 (0.47) ^h^	9.29 (0.40) ^g^	111.91 (1.53) ^a^	7.37 (0.19) ^g^
B1	83.16 (0.30) ^a^	−0.90 (0.13) ^d^	16.32 (0.45) ^d^	16.38 (0.41) ^d^	93.10 (0.52) ^f^	-
**Background shade C4**						
A3	76.21 (0.27) ^e^	0.03 (0.05) ^c^	26.75 (0.55) ^c^	26.75 (0.55) ^c^	89.92 (0.14) ^g^	11.93 (0.18) ^d^
OM 1.5	72.47 (1.34) ^g^	−2.46 (0.18) ^h^	10.70 (0.18) ^g^	10.97 (0.14) ^f^	102.97 (1.21) ^c^	15.26 (0.74) ^b^
OM 3.0	74.01 (0.68) ^f^	−2.74 (0.05) ^i^	10.47 (0.30) ^g^	10.82 (0.31) ^f^	104.61 (0.57) ^c^	16.16 (0.45) ^a^
BO 1.5	70.51 (0.64) ^h^	−1.09 (0.16) ^d^	10.91 (0.94) ^fg^	10.95 (0.93) ^f^	95.82 (1.41) ^e^	13.88 (0.94) ^c^
BO 3.0	72.05 (1.72) ^g^	−2.77 (0.17) ^ij^	7.82 (0.29) ^i^	8.29 (0.29) ^h^	109.56(1.31) ^b^	16.85 (0.71) ^a^
C4	61.58 (0.25) ^i^	1.30 (0) ^a^	34.20 (0.44) ^a^	27.36 (0.36) ^bc^	89.76 (0.05)	-

L*, Lightness; a*, green–red value; b*, blue–yellow value; C*, Chroma; H*, Hue. Same superscript letters within a column indicate no statistically significant differences (*p* > 0.05). Different letters indicate significant differences (*p* < 0.05).

**Table 3:** Translucency parameter (TP_00_) of the resin composites.

Resin Composite	TP_00_ Value
Control (Z350)	3.16 (0.15) ^c^
OM	8.70 (0.12) ^a^
BO	7.68 (0.08) ^b^

Different letters indicate significant differences (*p* < 0.05).

**Table 4:** Flexural strength and flexural modulus of the resin composites.

Resin Composite	Flexural Strength (MPa)	Flexural Modulus (GPa)
Control (Z350)	139.78 (25.19) ^a^	11.48 (1.74) ^A^
OM	108.90 (20.03) ^bc^	6.67 (1.70) ^B^
BO	124.01 (15.97) ^ab^	4.43 (0.57) ^C^

Different letters indicate significant differences (*p* < 0.05).

**Table 5:** Vickers microhardness of top and bottom surfaces and B/T ratio of the resin composites.

Resin Composite	Increment Depth	Top	Bottom	B/T Ratio (%)
Control (Z350)	2 mm	84.13 (1.19) ^b^	56.89 (3.70) ^a^	67.61 (4.03) ^c^
	4 mm	87.26 (2.66) ^a^	16.88 (2.18) ^e^	19.41 (2.86) ^d^
OM	2 mm	52.82 (1.11) ^d^	45.18 (4.26) ^b^	89.24 (3.96) ^a^
	4 mm	58.46 (2.07) ^c^	36.94 (3.66) ^d^	63.23 (6.21) ^c^
BO	2 mm	43.35 (0.64) ^f^	41.07 (0.85) ^c^	94.75 (2.64) ^a^
	4 mm	48.93 (4.87) ^e^	37.99 (3.09) ^cd^	78.69 (12.01) ^b^

Same superscript letters within a column indicate no statistically significant differences (*p* > 0.05). Different letters indicate significant differences (*p* < 0.05).

## Data Availability

The original contributions presented in the study are included in the article; further inquiries can be directed to the corresponding author.
